# Personalized Nutrition Therapy without Weight Loss Counseling Produces Weight Loss in Individuals with Prediabetes Who Are Overweight/Obese: A Randomized Controlled Trial

**DOI:** 10.3390/nu16142218

**Published:** 2024-07-11

**Authors:** Raedeh Basiri, Lawrence J. Cheskin

**Affiliations:** 1 Department of Nutrition and Food Studies, George Mason University, Fairfax, VA 22030, USA; 2 Institute for Biohealth Innovation, George Mason University, Fairfax, VA 22030, USA; 3 Department of Medicine, Johns Hopkins School of Medicine, Baltimore, MD 21205, USA

**Keywords:** prediabetes, personalized nutrition therapy, diet therapy, body composition, obesity, overweight, body composition, muscle mass, weight loss, diabetes, continuous glucose monitoring

## Abstract

Obesity stands out as a primary risk factor for diabetes. Attaining healthy weight loss, especially reducing body fat, is important in managing prediabetes and preventing progression to full diabetes and its co-morbidities. This study examined the effects of personalized nutrition therapy (PNT) combined with continuous glucose monitoring (CGM) on body weight and composition in individuals with prediabetes. A total of 30 individuals with prediabetes who were overweight or obese were assigned randomly to either the treatment, observed CGM data plus PNT, or the control group which was blinded to their blood glucose results throughout the study. Both groups were provided with dietary recommendations for calorie intake and macronutrient distribution, coupled with personalized goal setting for glucose control and healthy eating, without any specific emphasis on weight reduction or changes in physical activity. Regular visits were scheduled every 10 days to perform measurements and replace CGMs. Data were analyzed using General Linear Model with repeated measures. Over the 30-day follow-up period, both groups experienced significant reductions in weight and fat mass. The treatment group exhibited two-fold greater reductions in both weight and fat mass, a significant decrease in carbohydrate intake, and a significant increase in time spent on physical activitycompared to the control group. In addition, compliance was notably higher in the treatment group. These findings indicate that overweight or obese individuals with prediabetes can achieve weight loss and improved body composition through personalized education for glucose control, without exclusively emphasizing weight loss as the primary objective. Additionally, the real-time feedback provided by CGM enhances these improvements.

## 1. Introduction

The prevalence of prediabetes, diabetes, and related complications in the United States continues to increase. Recent reports from the Centers for Disease Control and Prevention (CDC) show that 38% of Americans have prediabetes [1]. Overweight and obesity are strong predictors of prediabetes, and if not addressed promptly, can progress to diabetes and diabetes-related complications [2,3].

Scientific evidence emphasizes the significance of modest weight loss in improving metabolic risk factors associated with obesity-related conditions like prediabetes [4]. Currently, medical recommendations emphasize restrictive diets as a means to achieve weight loss and improve insulin sensitivity; however, even when these approaches are initially successful, weight regain often occurs [5,6], posing renewed health risks [7]. Another important factor often overlooked is that weight loss may result in undesirable changes in body composition, such as a reduction in fat-free mass, which is associated with insulin resistance [8,9]. Maintaining lean body mass while adhering to a substantial caloric deficit is challenging, irrespective of the diet’s macronutrient distribution [10]. Hence, encouraging people to follow healthy eating behaviors and set realistic personalized goals could help with gradual and sustainable weight loss [11] and support favorable changes in body composition [12]. 

Body mass index (BMI) is a traditional measurement for evaluating a person’s body weight; however, studies have shown that the percentage and location of fat in the body are better predictors of insulin resistance [13,14]. Therefore, it is important to examine changes in body composition along with weight changes to better support healthy weight loss. 

It is also crucial to approach weight loss interventions with a focus on sustainability and personalized care to optimize health outcomes, particularly for those at high risk of developing full diabetes. In supporting individuals with prediabetes, real-time blood glucose monitoring devices (CGM) enable dietitians to tailor dietary recommendations to the individual’s physiological needs and personal preferences, facilitating the development of healthful eating behaviors and optimizing metabolic outcomes. Utilizing CGMs allows individuals with prediabetes to observe the positive effects of healthful eating on their blood glucose control in real-time and incentivizes sustainable behavior changes. 

In this study, energy and macronutrients were calculated and recommended to participants for the maintenance of their weight; there were no recommendations for following a calorie-restricted diet or increasing physical activity for weight loss. It was hypothesized that recommending healthy eating and personalizing recommendations based on individuals’ dietary preferences and their body’s glycemic reaction to different types of foods would increase compliance while improving body weight and body composition among these overweight and obese individuals with prediabetes. 

## 2. Materials and Methods

IRB approval was obtained from George Mason University and the study was registered on www.ClinicalTrials.gov (accessed on 2 November 2021, ID: NCT05161897, Approval Number: 1825534-2). Individuals aged 45–65 years of any race or ethnicity were screened via phone and in-person based on the inclusion and exclusion criteria. Participants were eligible if they were not diagnosed with diabetes before, their hemoglobin A1c (HbA1c) level at baseline was between 5.7% and 6.4%, and their BMI was between 25 and 39.9 kg/m^2^. 

People were excluded if they were pregnant or lactating, or if they had active cancer, thyroid, kidney, liver, or pancreatic disease. Also excluded from the study were the following criteria: heavy cigarette smokers (≥25 cigarettes a day), those who reported consuming >12 alcoholic drinks/week on average or had major dietary restrictions that, in view of the investigator, would limit the ability to deliver effective dietary interventions, those who were participating in any weight loss or dietary program, those taking prescribed appetite suppressants, or those who were participating in another investigational study. 

After obtaining informed consent, participants were randomized to the treatment or control group. Resting blood pressure, height, weight, and hip and waist circumference were evaluated at baseline, and medical and medication history was obtained. Participants completed a 24 h recall form for the day before baseline and each subsequent visit using the Automated Self-Administered 24-Hour (ASA24^®^) Dietary Assessment Tool [15]. Participants were also provided with a CGM device to be used for assessing continuous glucose concentrations and glycemic variability during the 30-day follow-up. 

The treatment group was able to see their blood glucose on their mobile devices in real-time, while the control group was blinded to these data. Both groups were asked to wear the CGM devices and follow their typical dietary intake and physical activity during the first 10 days of the study. After the first 10 days, both groups were asked to come back to replace their CGM and to receive personalized nutrition therapy. Visits with both groups were scheduled every 10 days for 30 days. During the second visit, both groups were provided with dietary recommendations by a dietitian based on their energy needs for maintaining participants’ current weight and recommended macronutrient distribution for individuals with diabetes [16]. The Mifflin–St Jeor equation [17] was used to calculate participants’ energy needs for weight maintenance, considering their sex, weight, height, age, and activity level. Moderate carbohydrate diets (50% carbohydrate, 20% protein, 30% fat) were prescribed, and participants were educated about different food groups, serving sizes, the recommended amount of carbohydrates for each meal, and the carbohydrate choices using the exchange list for diabetes [18]. The dietitian also asked about the typical dietary intake of participants and made recommendations based on their personal preferences. In the treatment group, the blood glucose data recorded in the CGM was also reviewed and discussed during each visit. Foods that elevated post-meal blood glucose levels above 140 mg/dL were identified as unfavorable for blood glucose control. Recommendations were provided to minimize or eliminate the consumption of these flagged foods to reduce the frequency of experiencing high blood glucose events. The dietitian continued to review the daily blood glucose data and dietary intake of participants in the treatment groups during visits three and four. This information was utilized to educate the treatment group and establish personalized goals for dietary modification to improve blood glucose control health outcomes. All participants underwent measurements, CGM replacement, and consultations with the dietitian during visits throughout the study. No specific instructions were provided regarding changes in physical activity for either group. The primary differences between the treatment and control groups were that the treatment group could view their blood glucose results in real-time, could receive personalized goals based on the CGM data, and their nutrition education was tailored accordingly. In contrast, both the dietitian and participants in the control group were blinded to the blood glucose values throughout the study.

### 2.1. Anthropometric Measurements 

Height without shoes was measured using a wall-mounted stadiometer, weight was assessed using a digital scale (Health o meter^®^ Professional Scales, McCook, IL, USA), and BMI was calculated using the formula: BMI = weight (kg)/[height (m)]^2^. Waist and hip circumferences were measured following National Health and Nutrition Examination (NHANES) guidelines [19]. Body composition was measured using InBody 270 (InBody Co., Ltd., Seoul, Republic of Korea). The InBody machine utilizes bioelectrical impedance analysis (BIA) to assess body composition. BIA operates by sending a small, alternating electrical current through the body and measuring the resistance encountered by the current. This resistance is then used to determine various body composition metrics, including body fat, water, lean mass, and other measurements [20].

### 2.2. Physical Activity Questionnaires

Although there was no specific emphasis on changing physical activity for participants, their physical activity levels were evaluated to assess any potential changes throughout the study. Physical activity patterns were assessed at baseline and the end of the study using the Five-City Project Physical Activity Recall [21], which evaluates leisure, occupational, and home activities. This tool was used to analyze the usual activity levels, consistency over time, and deviations from baseline. Research staff interviewed each participant at both time points, recording their physical activity over the past seven days using the Five-City Project Physical Activity Recall. 

### 2.3. Sample Size, Power Calculation, and Statistical Analysis

Using a randomized controlled design with two groups (treatment vs. control) and four time points (baseline and every 10 days until the end of the study [30 days]), the hypothesis that the intervention (PNT) would improve body composition was supported by a statistically significant interaction between time and intervention. For this study, power calculations were performed for a statistically significant interaction between the intervention (PNT vs. control) and time (four time points). The sample size was calculated based on an effect size of 0.25 (mean treatment difference and standard deviation) for the primary outcome measure (reduction in fat mass), which would be clinically relevant [22,23]. Using G*Power 3.1.9.4 statistical power analysis program [24], to have an 80% power to detect this difference with an alpha error of 0.05, a minimum of 24 individuals (i.e., 12 per group) was needed. Anticipating 20% missing data or dropout, we recruited a total of 30 individuals.

Data were analyzed using the Statistical Package for Social Science (SPSS) version 29.0 (SPSS, Inc., Chicago, IL, USA). The statistical significance threshold was set at *p* < 0.05 for all tests. Descriptive statistics were used to evaluate population characteristics, and an ANOVA table was employed to assess the distribution of covariates between the groups. General Linear Model with repeated measures was conducted to assess changes in dietary intake of energy and macronutrients, physical activity, and different measures of body composition. To determine the differences among groups, post hoc LSD correction for multiple comparisons was conducted. The effects of sex and age as potential covariates were analyzed, but these effects were not significant for the reported outcome measures. Consequently, the analysis was conducted without including covariates in the models.

## 3. Results

All 30 participants completed the study. The mean age (in years) of participants was significantly higher in the treatment (57.3 ± 5.2) versus the control group (52.7 ± 6.4). However, no significant differences were observed in the distribution of sex, BMI, ethnicity, and HbA1c between the groups. None of the participants had specific dietary restrictions or needed food assistance, and none were current cigarette smokers. Only one participant in the treatment and four participants in the control group had visited a registered dietitian (RD) in the past. The demographics of the participants are presented in Table 1.

### 3.1. Dietary Compliance

Dietary compliance was self-reported by participants. The average rate of compliance significantly increased in both groups during the study; however, it increased more substantially in the treatment group compared to the control group (16.3% to 92% vs. 14.2% to 74.3%; *p* < 0.001).

### 3.2. Dietary Intake of Participants

The average calorie intake of participants in the treatment group mildly changed from 1880 to 1785 kcal, while in the control group, it changed from 2059 to 2017 kcal. The mean percent calorie intake from protein numerically increased from 19.9% to 21.7% in the treatment group and remained the same at 19.46% in the control group. The mean percent calorie intake from fat increased from 36.7% to 40.1% in the treatment group and from 36.7% to 37.9% in the control group; however, these changes did not reach statistical significance in either group. The change in the mean percent calorie intake from carbohydrates was significant in the treatment group, decreasing from 44.8% to 38.8% (*p* = 0.03), but was not statistically significant in the control group, changing from 42.1% to 40.5%. The mean fiber intake numerically increased from 17.7 to 21 g in the treatment group and decreased from 23.8 to 17.7 g in the control group. Although the change in fiber intake was not statistically significant, it is clinically relevant.

### 3.3. Changes in Physical Activity

**Figure 1:**
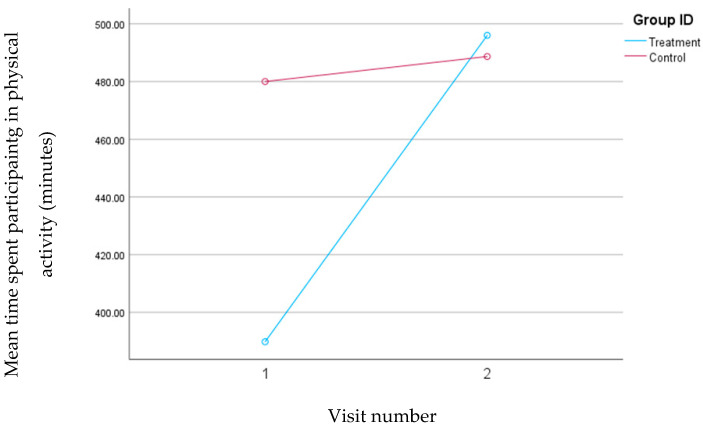
Changes in the mean time spent participating in physical activity (minutes) for the treatment and control groups during the 30-day follow-up period.

No significant differences were observed between the groups at baseline regarding physical activity. However, the mean time spent on physical activity numerically increased from 390 to 496 min in the treatment group and from 480 to 489 min in the control group. Although these changes did not reach statistical significance, the increase in the average time spent on physical activity was more pronounced in the treatment group compared to the control group, and it is clinically relevant. The changes in physical activity of the groups are outlined in Figure 1.

### 3.4. Changes in Anthropometric Measures

#### 3.4.1. Weight

**Figure 2:**
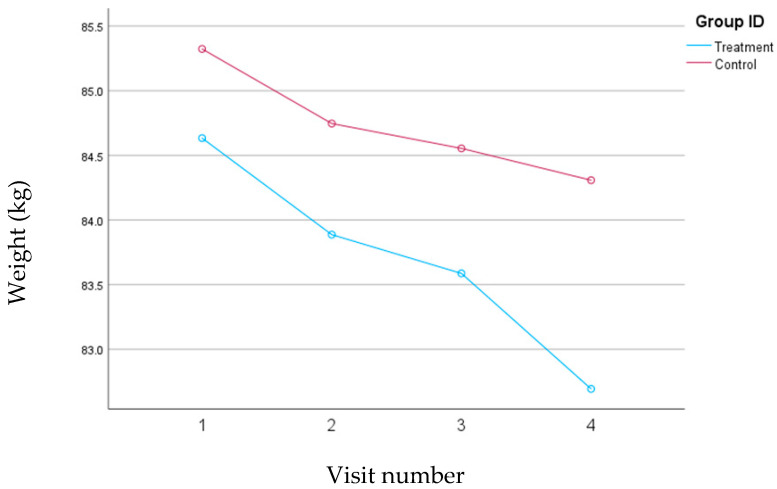
Changes in weight (kg) for the treatment and control groups during the 30-day follow-up period, with study visits scheduled every 10 days.

Both groups experienced significant weight loss during the study; however, the mean reduction in weight was almost double in the treatment group, 4.27 lbs. (*p* < 0.001), when compared to the control group, 2.22 lbs. (*p* = 0.01). The changes in weight for both groups are depicted in Figure 2.

#### 3.4.2. Body Mass Index (BMI)

**Figure 3:**
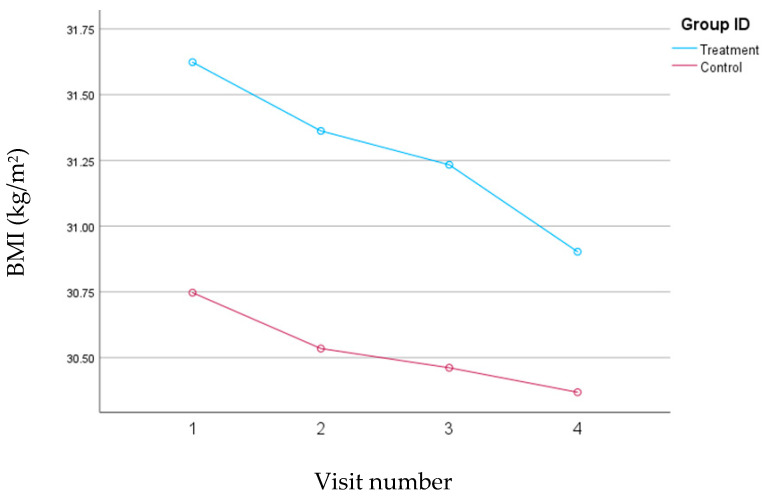
Changes in BMI (kg/m^2^) for the treatment and control groups during the 30-day follow-up period, with study visits scheduled every 10 days.

At baseline, the mean BMI was slightly higher in the treatment group (mean ± SD: 31.6 ± 4.50 kg/m^2^) compared to the control group (mean ± SD: 30.75 ± 4.11 kg/m^2^); this difference was not statistically significant. Both groups exhibited a significant reduction in BMI over the course of the study; however, the treatment group sustained a greater mean BMI decrease of 0.72 kg/m^2^ (*p* < 0.001) versus 0.38 in the control group (*p* = 0.01). The changes in BMI for both groups over the course of the study are depicted in Figure 3.

#### 3.4.3. Changes in Waist-to-Hip Ratio (W/H)

At the onset of the study, the waist-to-hip ratio (W/H) was slightly higher in the treatment group compared to the control group (mean ± SD: 0.93 ± 0.07 versus 0.90 ± 0.05, respectively). However, there were no significant changes observed in the W/H ratio in either group throughout the study period.

### 3.5. Changes in Body Fat Mass

At baseline, the mean body fat mass was higher in the treatment group (mean ± SD: 79.5 ± 20.68 lb.) compared to the control group (mean ± SD: 73.7 ± 22.24 lb.). The treatment group exhibited a significant fat loss of 2.4 lbs. (*p* = 0.002), whereas the control group showed a numerical decrease of 1.1 lbs., which did not reach statistical significance. Although the changes in body fat mass in the control group were not statistically significant, they have clinical relevance.

### 3.6. Changes in Skeletal Muscle Mass (SMM)

At the start of the study, the mean skeletal muscle mass was lower in the treatment group (mean ± SD: 58.89 ± 15.69 lbs.) compared to the control group (mean ± SD: 63.54 ± 13.57 lbs.). During the study, both groups experienced a notable decrease in SMM. The SMM in the treatment group was reduced by 1.06 lbs. (*p* < 0.001), and in the control group by 0.68 lb. (*p* = 0.02). However, there was no statistically significant difference between the two groups regarding the loss of SMM. 

### 3.7. Changes in Dry Lean Mass (DLM)

At baseline, the mean dry lean mass was lower in the treatment compared to the control group (mean ± SD: 28.45 ± 6.86 vs. 30.55 ± 6.14 lbs.). Both the treatment and control groups demonstrated significant reductions in DLM during the study (0.48 lb., *p* < 0.001 and 0.37 lb., *p* = 0.02, respectively). However, the mean reduction in DLM did not significantly differ between the groups. 

### 3.8. Changes in Total Body Water (TBW)

The mean TBW was higher in the control group (mean ± SD: 38.29 ± 7.29 l.) compared to the treatment group (mean ± SD: 35.55 ± 8.8 l.) at baseline. Both the treatment and control groups experienced significant reductions in TBW during the study; however, the reduction in TBW was more pronounced in the treatment group compared to the control group [0.62 l. (*p* < 0.01) vs. 0.34 l. (*p* = 0.01)].

## 4. Discussion

Our findings demonstrate that, overall, personalized nutrition therapy is effective for improving weight loss and reducing fat mass in overweight and obese individuals with prediabetes, regardless of whether or not participants were able to see the blood glucose results in real-time using CGMs. Of note, though, enabling participants to observe the immediate effects of dietary changes on their blood glucose levels enhanced compliance and the intervention’s effectiveness. Furthermore, utilizing CGMs as a behavior modification tool minimized unnecessary dietary restrictions. Thus, those individuals had greater dietary flexibility, which could be another reason for the observed increased compliance in the treatment group. 

Another potential reason for participants’ weight loss could be food journaling and calorie counting, which was part of the intervention for both groups. It has been shown that self-monitoring for weight loss, including food journaling or calorie counting, is an effective strategy for weight loss attempts [25]. 

The significant reduction in the dietary intake of digestible carbohydrates and the more substantial increase in the percentage of time spent participating in physical activity in the treatment group compared to the control group could also explain the more pronounced changes observed in weight and fat loss in the treatment group. Although no intervention was conducted to promote physical activity for either group during the study, observing blood glucose in real-time could have incentivized the treatment group to significantly enhance their physical activity. This highlights the importance of CGM use as a behavioral change tool in this population.

On average, the treatment group lost more skeletal muscle mass than the control group, which could be explained by a greater weight loss and total body water in the treatment group. These findings underscore the importance of emphasizing hydration when providing nutrition education and incorporating resistance training in weight loss programs. No significant changes were observed in the waist-to-hip ratio in either group, despite the significant weight loss and changes in body composition, which might be due to the short duration of the study. 

For individuals with prediabetes, CGM allows them and the dietitian to see the effects of having different types and amounts of foods on their blood glucose in real-time, which takes much of the tracking effort and risk of avoidance out of the equation. By tracking glucose trends and fluctuations, CGM devices offer actionable data that can help individuals and dietitians make informed decisions about their diet and optimize their nutrition to support weight loss and improve body composition. Utilizing CGM can also help people refrain from eating foods that are more likely to contribute to weight gain (such as French fries, potatoes, potato chips, sugary drinks, and refined grains) when they see how these foods result in blood sugar spikes [26]. Observing the positive effects of foods that support both weight loss and blood glucose control encourages them to consume these foods preferentially. These effects on dietary behaviors may explain our finding that the treatment group lost twice as much weight and fat mass as the control group. Our results align with a study by Ehrhardt et al., where 87% of CGM users reported modifying their food choices based on CGM data [27]. 

To our knowledge, this is the first study that evaluates the effects of personalized nutrition therapy along with using CGM on body composition in individuals with prediabetes. The diverse population included in this study contributes to the generalizability of the results. The significant changes observed in body weight and fat mass underscore the effectiveness of this approach in individuals with prediabetes who are overweight or obese, even with a relatively short period of follow-up. 

It has been shown that an increase in fat mass is a strong predictor of overall cardiometabolic risk [3] and the percentage of time spent in normal blood glucose levels (time-in-range) in patients with diabetes [28], and that there is a direct association between higher fat mass and serum glucose and HbA1c levels in individuals with prediabetes [29]. Thus, interventions that target reducing fat mass in this population are likely to produce significant health benefits.

A loss of muscle mass during weight loss is an inevitable side effect of weight loss [30]. Our participants also experienced a decrease in skeletal muscle mass (SMM) and dry lean mass (DLM). The observed SMM loss was proportional to the amount of weight loss. Thus, the non-significantly higher reduction in muscle mass observed in the treatment group compared to the control group was likely due to the more pronounced weight loss and the greater loss of total body water observed in this group. It is crucial to minimize the loss of muscle mass during weight loss programs to reduce its negative effects on health [31]. Studies suggest that a higher percentage of protein intake and incorporating physical activity can decrease muscle loss during weight loss [31,32]. The dietary recommendations given to participants in this study targeted a high intake of high bioavailability protein; however, as this study focused on dietary changes, no intervention regarding physical activity was implemented. Future research should aim to explore the optimal combination of protein intake and physical activity regimens to maximize the preservation of muscle mass in individuals with prediabetes.

This study is limited by its relatively small sample size and short follow-up period. Hence, the long-term effects of the intervention on body composition and its sustainability should be investigated in future studies. 

In summary, this study demonstrated the positive effects of the intervention on weight loss and reduction in fat mass in individuals with prediabetes. While the intervention showed promising outcomes in terms of improving body composition, future studies should focus on strategies to mitigate the loss of muscle mass while promoting weight loss in this population. Additionally, long-term follow-up studies are needed to assess the sustainability of these interventions and their impact on overall health outcomes in individuals with prediabetes.

## 5. Conclusions

Personalized nutrition therapy, emphasizing healthy eating without a primary focus on weight loss, coupled with the utilization of CGM devices, enhances weight loss and reduces fat mass in overweight and obese individuals with prediabetes.

## Figures and Tables

**Table 1:** Demographics of participants and distribution of covariates in the treatment and control groups.

Group	Treatment (*n* = 15)	Control (*n* = 15)	*p*-Value
Age (Mean ± SD)	57.3 ± 5.2	52.7 ± 6.4	0.04
Sex (Female/Male)	11/4	11/4	1.00
Race (White/Black/Asian)	9/1/5	10/2/3	
HbA1c%	5.7 ± 0.78	6.0 ± 0.2	0.26
BMI * (kg/m^2^)	31.8 ± 4.3	31.4 ± 4.5	0.84
Live alone (y/no)	1/14	2/13	0.55
Have medical insurance (y/no)	14/1	11/5	0.07
Employed (y/no)	11/4	12/3	0.7
Have financial concerns (y/no)	2/13	1/14	0.56
Visited RD ** in the past	1/14	4/11	0.64

* Body mass index. ** Registered dietitian.

## Data Availability

The raw data supporting the conclusions of this article will be made available by the authors upon request.
